# Digitally Guided Hydraulic Crestal Sinus Floor Elevation Versus Free‐Hand Osteotome Technique: A Single‐Blinded Randomized Controlled Clinical Trial

**DOI:** 10.1111/clr.70062

**Published:** 2025-10-28

**Authors:** Mahsa Enssi, Mohammadreza Karimi, Ardavan Etemadi, Mahsa Khademi, Hamoun Sabri

**Affiliations:** ^1^ Department of Periodontics, Faculty of Dentistry Tehran Medical Sciences, Islamic Azad University Tehran Iran; ^2^ Department of Radiology, Faculty of Dentistry Tehran Medical Sciences, Islamic Azad University Tehran Iran; ^3^ Department of Periodontics and Oral Medicine University of Michigan School of Dentistry Ann Arbor Michigan USA

**Keywords:** computer‐assisted surgery, dental implants, digital dentistry, guided surgery, maxilla, sinus floor augmentation, surgical flaps

## Abstract

**Objectives:**

To compare the clinical and radiographic outcomes of crestal sinus floor elevation (SFE) using a digital surgical guide (DSG) and hydraulic sinus lift technique versus the conventional osteotome technique.

**Methods:**

Patients requiring crestal SFE and single‐implant placement in the posterior maxilla with a minimum of 5 mm of residual bone height were included and randomly assigned to two groups of crestal SFE with (i) the conventional technique (elevation of a mucoperiosteal flap and SFE by the osteotome technique, control group) and (ii) by using a DSG (without flap elevation, by using the sinus lift hydraulic system in the presence of a DSG, test group). Frequency of intraoperative membrane perforation, surgical time, and patient‐reported outcomes were collected. Crestal bone loss at 6 months, new bone formation in the sinus, and implant position were assessed.

**Results:**

Twenty‐three patients (24 implants, 12 in each group) were included. The DSG group experienced significantly lower postoperative pain (measured by the number of analgesics taken, 1.67 ± 0.77 vs. 2.75 ± 1.21, *p* = 0.028), shorter surgical time (22.3 ± 2.4 vs. 33.1 ± 4.1 min, *p* < 0.001), and smaller deviation of implant angulation from the ideal position (2.9° ± 0.6° vs. 8.6° ± 1.3°, *p* < 0.001) compared to the conventional group. The difference in other parameters was not significant.

**Conclusion:**

Considering the study limitations, using a DSG with a hydraulic system for crestal SFE was superior to the conventional technique regarding pain score, surgical time, and implant angulation. Due to the relatively small sample size and lack of statistically significant differences in some parameters, these findings should be interpreted with caution.

**Trial Registration:**

Iranian Registry of Clinical Trials: IRCT20230529058333N1

## 1 Introduction

Edentulism of the posterior maxilla can cause problems such as difficult mastication and pneumatization of the maxillary sinus towards the edentulous ridge (Attanasio et al. 2020). Pneumatization of the maxillary sinus following tooth loss reduces the available vertical bone height in the posterior maxilla, which can complicate the placement of implants with ideal length (Saleh et al. 2024).

Moreover, bone density in the posterior maxilla negatively affects implant survival. Sinus floor elevation (SFE) is indicated for such patients; otherwise, bone resorption and pneumatization of the maxillary sinus would continue, decreasing the chance for optimal oral rehabilitation (Bernardello et al. 2011).

Different treatment plans have been proposed for implant placement in an atrophic edentulous posterior maxilla, such as the placement of short or angulated implants and SFE. Placement of short and angulated implants is challenging due to low bone density (Pozzi and Moy 2014). Thus, SFE is often preferred. Several methods may be adopted for SFE, such as the lateral window technique and the crestal approach. The main indication of the crestal SFE is the absence of sufficient bone height in the posterior maxilla for implant placement (Elian and Barakat 2018). The 5‐year success rate of SFE is reportedly 96% when the residual crestal bone height is 5 mm or higher; this rate decreases to approximately 85% when the residual bone height is ≤ 4 mm (Rosen et al. 1999). The crestal approach is a minimally invasive approach with fewer complications compared to SFE by the lateral window approach. The latter is more invasive and requires a much larger surgical field. However, the main disadvantage of the crestal approach is the risk of intraoperative sinus membrane perforation due to limited vision (Pjetursson et al. 2009). Another crestal technique was introduced by Summers (Summers 1994) in 1994. However, benign paroxysmal positional vertigo is among the most common complications of Summers' osteotome technique that occurs as the result of the hammering effect of osteotomes used during the sinus surgery (Baloh et al. 1987). Such complications encouraged researchers to seek alternative techniques with less complications and led to the introduction of techniques involving the use of kits with safe drills for accessing the sinus; the SFE hydraulic system is among such techniques (Sotirakis and Gonshor 2005; Chen and Cha 2005).

The SFE hydraulic system, introduced by Chen and Cha (2005), is a suggested technique for safer lifting of the sinus membrane. It includes a fluid‐based system that can apply a uniformly distributed hydraulic pressure on the Schneiderian membrane along its entire thickness (Vitkov et al. 2005).

Achieving the best implant position three‐dimensionally is an important long‐term success criterion for implant treatments since it can guarantee a suitable prosthetic restoration design (Meneghetti et al. 2023). Optimal implant positioning can result in more favorable prosthetic outcomes, including improved function, esthetics, and long‐term success. Moreover, correct implant position is imperative to ensure long‐term durability and optimal access for oral hygiene maintenance (Smitkarn et al. 2019).

Digital surgical guides (DSGs) are fabricated, mainly with the help of cone‐beam computed tomography (CBCT) and dental arch scanning and are increasingly used for oral surgical procedures (Kernen et al. 2020). DSGs enable crestal SFE and subsequent implant placement with higher precision, compared with the conventional technique. Also, they enable less invasive procedures, that is, flapless surgery (Fornell et al. 2012). Evidence shows that flapless surgery has several advantages including reduced postoperative pain and discomfort, as well as less crestal bone loss (Frizzera et al. 2021; Lindeboom and Van Wijk 2010). However, some factors may compromise the accuracy of DSGs, and gaps in knowledge regarding their efficacy remain (Derksen et al. 2019).

There is an ongoing debate regarding a minimally invasive technique for SFE and implant placement with minimal complications and most favorable results. Considering the scarcity of studies comparing the use of DSGs with the conventional technique, this study aimed to compare the clinical and radiographic results, as well as patient reported outcomes (PROs), including postoperative pain and discomfort of crestal SFE with a hydraulic SFE system in presence of a DSG (flapless technique) with the conventional technique (osteotome and mallet) in patients with an edentulous posterior maxilla.

## 2 Materials and Methods

### 2.1 Experimental Design and Ethical Aspects

This study was conceptualized as a single‐blinded randomized controlled clinical trial where one group underwent crestal SFE with a hydraulic system in the presence of a DSG (flapless technique) and the other group underwent the conventional technique using an osteotome and mallet. The results were reported in accordance with the Consolidated Standards of Reporting Trials (CONSORT) (Schulz et al. 2010).

The study was conducted at the Periodontology Department of the Faculty of Dentistry, Tehran Medical Sciences, Islamic Azad University. The study protocol was approved by the ethics committee of the university (IR.IAU.DENTAL.REC.1401.023) and registered in the Iranian Registry of Clinical Trials (IRCT20230529058333N1; registration date: 06‐28‐2023; start date: 07‐07‐2023). A signed informed consent was obtained from all participants at the screening phase, and the trial was conducted in full accordance with the principles outlined in the Declaration of Helsinki (World Medical Association 2013).

### 2.2 Study Population, Eligibility Criteria, and Settings

The inclusion criteria were the missing of one or two teeth in the premolar or molar region of the maxilla, a minimum of 5 and a maximum of 7 mm residual bone height at the edentulous area. Residual bone height was measured on preoperative CBCT scans using calibrated measurement tools within the Romexis software (Planmeca, Finland). Measurements were performed in the sagittal plane at the center of the edentulous span, defined as the midpoint between the adjacent teeth, with the vertical distance calculated from the alveolar crest to the sinus floor.

All included implant sites were fully healed ridges, with a minimum of 4 months of healing following tooth extraction. No ridge preservation or grafting procedures were performed at the time of tooth extraction. Periodontal health was characterized by no bleeding on probing and O'Leary's plaque index < 25% (O'Leary 1972).

The exclusion criteria were systemic diseases contraindicating oral surgical procedures such as uncontrolled diabetes mellitus, cerebrovascular accident, recent myocardial infarction, and severe coagulopathies; infection of the surgical site or adjacent teeth; acute sinusitis; presence of pathological or large cystic lesions; chronic sinusitis occupying a large sinus space; pregnancy; age under 20 years; substance or alcohol abuse; history of head and neck radiotherapy or chemotherapy in the past 2 years; smoking more than 10 cigarettes/day; extensive edentulism causing vertical dimension loss; and oblique maxillary sinus floor with an angulation > 45°.

### 2.3 Outcomes

The occurrence of sinus membrane perforation during the crestal sinus floor elevation procedure was initially considered the basis for sample size calculation (Elian and Barakat 2018).

1. The analyzed primary outcomes were radiographic bone gain and patient‐reported outcomes (PROs), including surgical operation time and postoperative pain.
2. The secondary outcomes included changes in crestal bone levels and the accuracy of implant placement in terms of buccolingual angulation and mesiodistal positioning (distance from the ideal implant shoulder line).

### 2.4 Treatment Allocation, Allocation Concealment and Blinding

Each eligible patient was randomly assigned to receive either digitally guided flapless or conventional crestal SFE according to a computer‐generated randomization list. Block randomization was performed to ensure an equal distribution of the number of procedures performed per patient among treatment groups. The assignment of eligible patients to treatment was recorded using sealed envelopes and was disclosed to each clinical operator at the end of the screening appointment. Moreover, the radiologist who assessed the radiographs and the clinician who assessed the outcomes were blinded to the group allocation of patients, and the study had a single‐blinded design.

### 2.5 Interventions

#### 2.5.1 Pre‐Surgical Phase

After obtaining written informed consent from the patients, a CBCT scan (Planmeca, Finland) was obtained from the edentulous region with a 200 μm voxel size, 11 × 8 cm field of view, 90 kV tube potential, and 9 mA tube current for the fabrication of a DSG and selection of implant size (Pozzi et al. 2011). Implant size was selected in all patients according to their CBCT scan and taking into account 2–3 mm of sinus floor elevation for the purpose of standardization. To determine the required magnitude of SFE, the primary available bone of each patient was first quantified according to their CBCT scan, and then implant height was determined such that by subtracting it from the primary bone thickness, the required amount of lifting was calculated to be 2–3 mm. The patients were then randomly assigned to two groups (*n* = 12) for crestal SFE (i) with the conventional method (elevation of a mucoperiosteal flap and sinus lifting by the osteotome technique) and (ii) by using a DSG (without flap elevation, by using the SFE hydraulic system in the presence of a DSG).

#### 2.5.2 Fabrication of DSG

**FIGURE 1:**
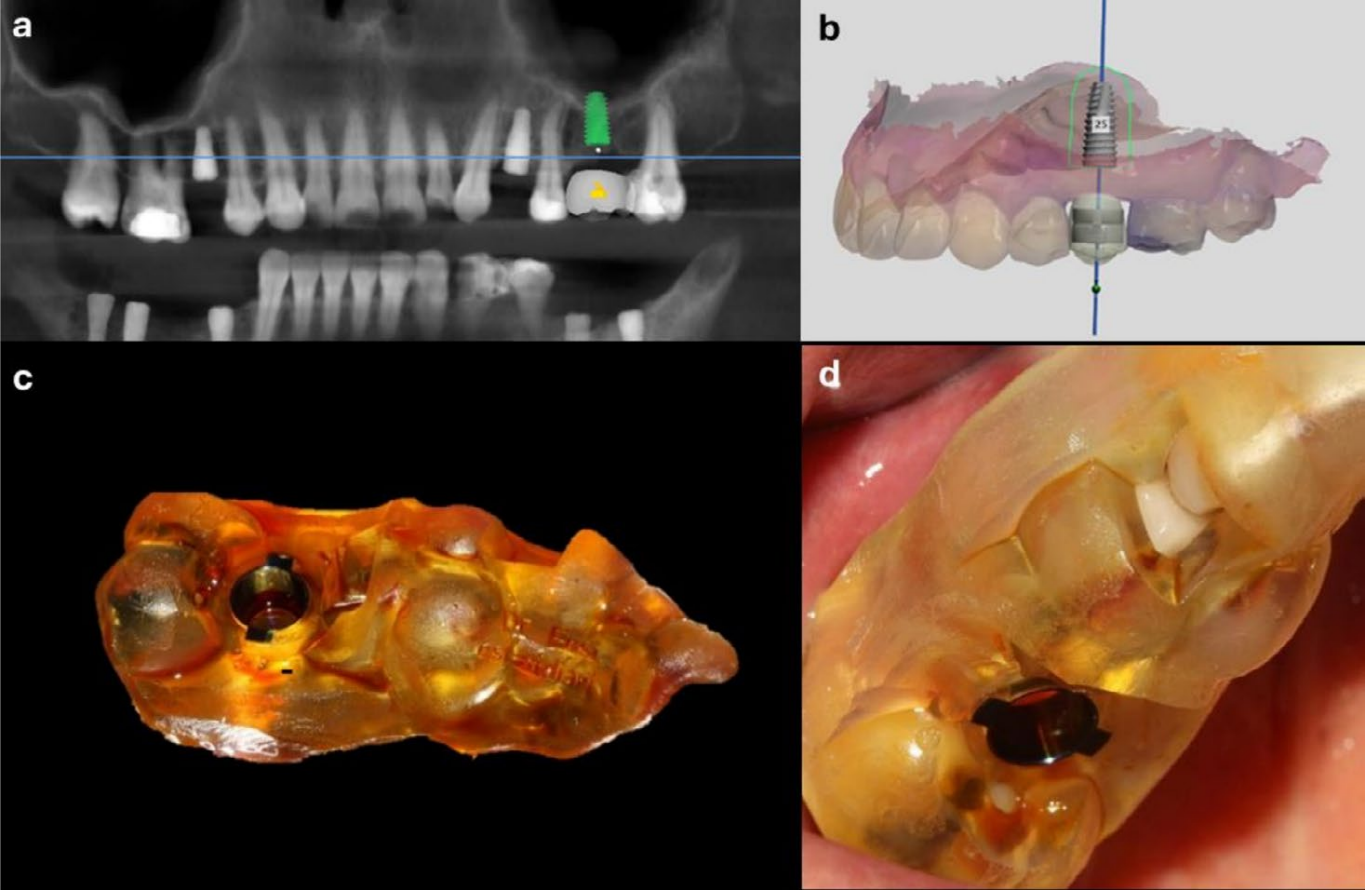
(a, b) Designing the implant position for fabrication of a DSG, (c, d) fabricated DSG and try‐in of DSG prior to surgery.

From the patients in the DSG group, an intraoral scan (Intraoral Carestream Scanner 3700, USA) was obtained, and the corresponding CBCT and intraoral scan data were combined in the ImplantStudio software program (Attanasio et al. 2020; Pozzi et al. 2011). As mentioned earlier, the required amount of SFE was considered to be 2–3 mm, and the best implant orientation regarding angulation and distance (by taking into account the final position of prosthetic restoration) was determined by the surgeon. Mesiodistally, the implant was oriented parallel to the adjacent mesial tooth. Buccolingually, the implant was oriented according to the functional cusp of the opposing tooth. Mesiodistally, the implant was positioned at the midpoint of the edentulous area. Buccopalatally, it was positioned such that a minimum of 1.5 mm of buccal bone and a minimum of 1 mm of palatal bone remained. To determine the required osteotomy depth, cross‐sectional images of the surgical site were required. Also, the distance between the tip of the crestal bone and the sinus floor was measured. The DSG was designed by the software accordingly. It was dentally supported by the adjacent teeth and was a fully guided type (presence of guide during the entire phases of drilling and implant placement with predetermined angulation) (Figure 1) (Attanasio et al. 2020). The DSG was printed by a printer (Phrozen Printer Sonic Mini 4k, Taiwan) using polymethyl methacrylate and had titanium sleeves (Dio, South Korea) according to the determined position and angulation of dental implants. The internal diameter of sleeves matched the diameter of the drills and connectors present in the implant surgical kit (Attanasio et al. 2020).

#### 2.5.3 Surgical Process

**FIGURE 2:**
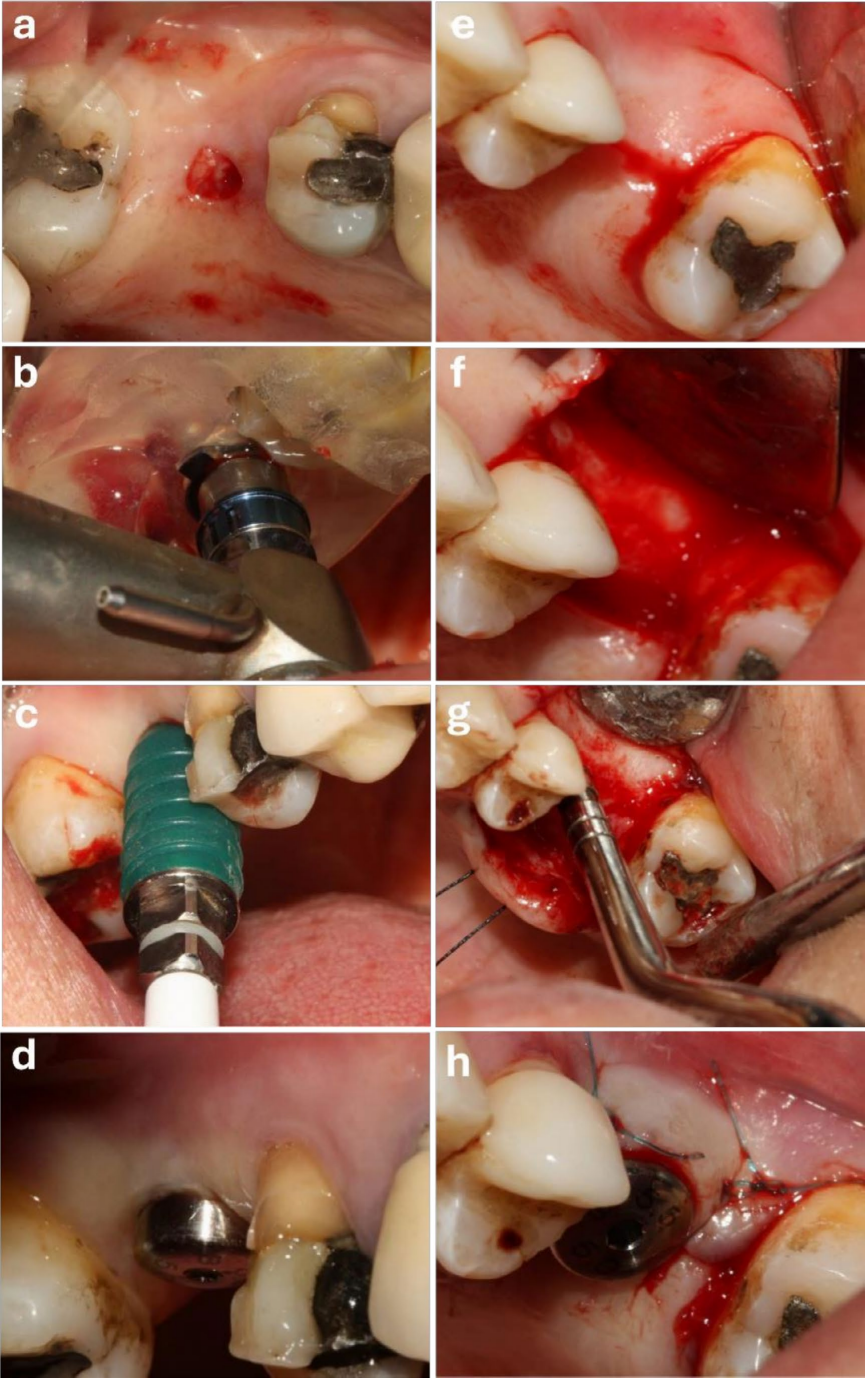
(a, b) Surgical procedure in the presence of DSG (tissue punch and drilling), (c) sinus lift via hydraulic system, (d) healing abutment in the guided group (no need for suture), (e, f) mid crestal incision and full‐thickness muccoperiostal flap elevation, (g) sinus lift (osteotome technique) and (h) healing abutment in the conventional group.

All patients were asked to rinse 0.2% chlorhexidine mouthwash for 1 min. Buccal and palatal infiltration anesthesia was induced by injection of one or two cartridges of 2% lidocaine plus 1:80,000 epinephrine (Darupakhsh, Iran) (Pjetursson and Lang 2014). The patients were then randomly assigned to two groups (*n* = 12). Figure 2 depicts surgical steps in both groups.

- *Conventional group*: A #15 surgical scalpel was used to make an incision at the midpoint of the edentulous ridge crest, and a full thickness mucoperiosteal flap was elevated. According to the CBCT measurements, the primary implant site was drilled by the pilot drill of the implant surgical kit (DIOnavi Master Kit, Dio, South Korea). Next, a sequence of drills from pilot to final were used for preparation of implant osteotomy site, which was extended to 1–2 mm below the sinus floor (Pjetursson et al. 2009). The first osteotome (Dentium Osteotome Kit, South Korea) which is tapered was used with mild tapping by a mallet to compress the sinus floor. After reaching the sinus floor, the osteotome was entered by 1 mm beyond the prepared length by mild tapping to induce a greenstick fracture in compacted bone of the sinus floor. Next, the second tapered osteotome, which has a larger diameter than the first one was introduced into the site to the same length as the first osteotome and further expanded the induced fracture in the sinus floor. The final osteotome used had a diameter 1–1.5 mm smaller than the implant diameter (Pjetursson et al. 2009).
- *DSG group*: Prior to surgery, the DSG was tried‐in to ensure its optimal fit (Figure 1d). Next, in presence of DSG, tissue was punched out using the punch tool available in the kit (DIOnavi Master Kit, Dio, South Korea) (Figure 2a). In presence of the DSG, the DIOnavi Master pilot drill (Kit, Dio, South Korea) was used for cortical bone penetration. The next drills were then used according to the protocol (Figure 2b) (Attanasio et al. 2020; Pozzi and Moy 2014). The drills of the crestal sinus lift kit (DIOnavi Flapless Crestal Sinus Kit, South Korea) were used according to instructions with proper stoppers to reach the final length and access the sinus floor. After accessing the Schneiderian membrane, it was gently elevated by using 5 cc of saline and the connector of the HydroLift system (Bruschi et al. 2021).

For the purpose of standardization of patients, implant length was selected such that 2–3 mm of lifting was required; no bone graft materials or substitutes were used in either group. The sinus was elevated without grafting, and implants were placed simultaneously.

Immediately after completion of the procedure in both groups, the occurrence of sinus membrane perforation was evaluated by the Valsalva maneuver or the nose blowing test. For this purpose, the nostrils of the patient were closed, and the patient was asked to blow into their nose (Pjetursson et al. 2009). Air leakage through the implant site indicated the occurrence of perforation. In the case of intraoperative sinus membrane perforation, the case was considered an immediate failure and excluded from further follow‐up. To maintain the planned sample size for the analysis of secondary outcomes, additional subjects were recruited and randomly allocated.

Dental implant (Dio UF II Implant, South Korea) was then placed. For the purpose of standardization, in all implants, the healing abutment was tightened only in case of the presence of optimal (30 N/cm) torque. Torque was measured using a calibrated manual torque wrench and recorded in Ncm.

The surgical site was sutured with 5‐0 Nylon suture (Supalon) in patients with flap surgery (Attanasio et al. 2020; Pjetursson et al. 2009; Becker et al. 2009).

Surgical time (duration of procedure) was recorded for all patients. Antibiotics (500 mg amoxicillin three times/day for 5–7 days), 400 mg ibuprofen as an analgesic and anti‐inflammatory drug, and 0.2% chlorhexidine rinse twice a day for 1 week were prescribed for patients. The patients were instructed to use analgesics only in case of pain (Attanasio et al. 2020; Pjetursson et al. 2009; Becker et al. 2009).

After surgery, parallel periapical radiographs were obtained by a Digora Optime scanner (Planmeca, Finland) with 60–70 kVp tube potential, 6 mA tube current, 0.04 s time, and a PSP sensor with 0.035 mm pixel size, 14 bit, and 14.3 Ip/mm resolution. This radiograph served as the reference radiograph for later measurements of crestal bone. One week after surgery, the patients were recalled to assess their healing process and also for suture removal. The patients were then asked about their pain and the number of analgesics taken by a blinded interviewer. Six months after surgery, the patients underwent radiographic examination again, and parallel periapical radiographs were taken by the same position and exposure settings as the reference radiographs taken immediately after surgery to assess the changes in crestal bone. The distance between the bone crest and the uppermost surface of the fixture (implant platform) was measured on both reference and 6‐month radiographs using Scanora software, and their difference was reported as the change in crestal bone (Si et al. 2013). A CBCT was also obtained to assess the maxillary sinus radiographical bone fill and implant position. The amount of newly formed bone in the sinus was quantified by assessing the baseline and 6‐month CBCT scans using Romexis software. For this purpose, the axial plane was passed through the coronal third of the roots, and the sagittal plane was oriented such that it passed through the middle of the roots of the adjacent teeth, yielding an oblique sagittal view (Figure S1). A fixed reference point was considered for the purpose of comparison after 6 months so that the changes caused by bone loss would not affect the measurement of the newly formed bone in the sinus. This reference was a line passing from the highest point of the adjacent tooth crown. On the preoperative CBCT scan, a line was drawn at the midpoint of the edentulous area perpendicular to the reference line and extended to the sinus floor, and the obtained value was calculated (Figure S1). On the 6‐month CBCT scan, a line passing through the implant center was drawn perpendicular to the reference line (Figure S1). The difference between these numbers indicated the amount of newly formed bone within the sinus (Suk‐Arj et al. 2019). To ensure standardization of the groups, both the initial vertical bone height and the magnitude of sinus lifting were compared between the two groups.

The ideal mesiodistal angulation of the implant was determined as parallel to the longitudinal axis of the adjacent mesial tooth and was marked on CBCT scans by Romexis software. Also, the ideal distance of the implant shoulder from the adjacent structures was determined on the CBCT scan. Next, to assess the implant position, deviations in implant angulation and distance from the ideal position were evaluated (Figure S1) (Tang et al. 2019). All radiographs were evaluated by a radiologist blinded to the group allocation (surgical technique) of patients. Radiologists reevaluated a random sample of radiographs at two separate time intervals to assess intra‐examiner reliability, and the intraclass correlation coefficient (ICC) showed excellent (> 0.9) measurement consistency. All patients were referred for prosthetic treatment after 6 months for the purpose of standardization and considering their optimal bone density.

### 2.6 Sample Size Calculation

The sample size was calculated based on the expected difference in sinus membrane perforation rates between groups, as reported in a previous clinical study by Elian and Barakat (2018). In their study, the perforation rate observed during crestal sinus floor elevation was approximately 16.7%, with 2 out of 12 patients experiencing minor membrane perforations. Based on this reference, we performed a two‐sample comparison of proportions using PASS 11 software, assuming an *α* of 0.05, a power of 80% (*β* = 0.2), and a clinically meaningful difference of 25% in perforation rates between the groups. The estimated minimum sample size was 12 per group (total *N* = 24).

However, it should be noted that perforated cases were excluded per protocol due to the inability to proceed with the assigned surgical technique in the hydraulic group. Therefore, perforation was not analyzed as a study outcome, and the final analyzed primary outcomes were radiographic bone gain and patient‐reported outcomes.

### 2.7 Examiner Calibration

To assess intra‐examiner reproducibility, radiographic measurements were repeated on five non‐study CBCT cases by the same examiner at two time points, 2 weeks apart. The intraclass correlation coefficients (ICCs) demonstrated excellent agreement: 0.94 for residual bone height, 0.91 for sinus lift measurements, and 0.89 for crestal bone level changes.

### 2.8 Statistical Analysis

Data were analyzed using SPSS version 26 (SPSS Inc., IL, USA). Normal distribution of data was evaluated by the Shapiro–Wilk test. Accordingly, the Pearson chi‐squared test was used to compare gender distribution, while an independent sample *t*‐test was applied to compare the mean age between the two groups. The Fisher's exact test was used to compare the frequency of membrane perforation. Due to non‐normal distribution of surgical time, number of taken analgesics, implant angulation, initial vertical bone height, and magnitude of sinus lifting, these were compared between the two groups using the Mann–Whitney test. Mesiodistal distance, mesial and distal bone loss, and new bone formation in the sinus had a normal distribution and were compared using independent samples *t*‐test. A sensitivity analysis was performed by excluding the single patient who received two implants to account for potential clustering. The level of statistical significance was set at 0.05.

## 3 Results

### 3.1 Study Population

**FIGURE 3:**
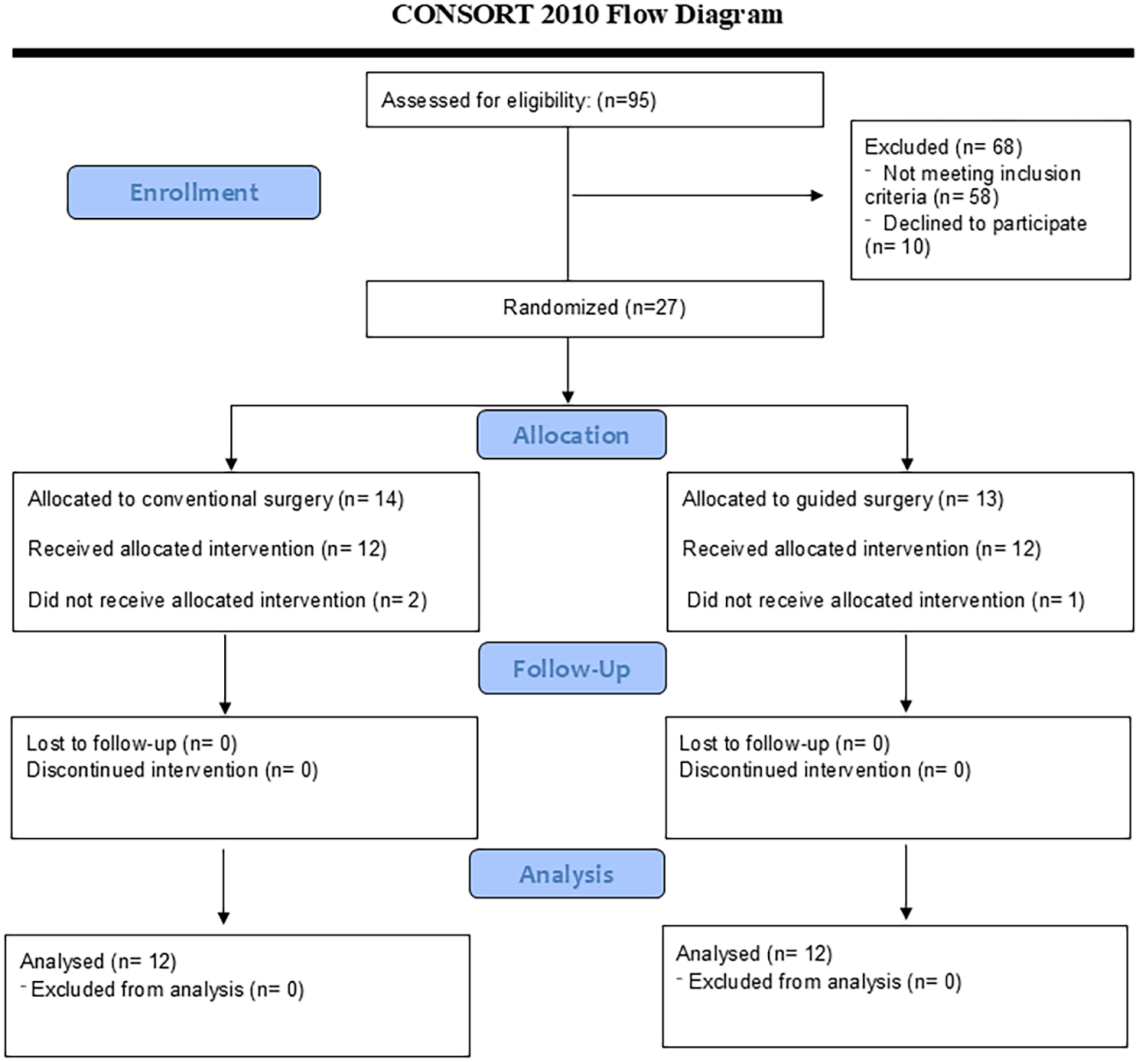
CONSORT 2010 flow‐diagram of patient selection and allocation.

The sample consisted of 23 patients (24 implants, with one female patient requiring two implants—one with conventional and the other through DSG), including 10 females. The mean age was 39.84 ± 2.83 years (range 25–51 years) in the conventional group and 41.33 ± 3.09 years (range 27–48 years) in the DSG group. The difference in gender distribution and mean age difference was not significant between the two groups (*p* = 0.162 and 0.800, respectively). Three patients experienced membrane perforation during the surgical process; out of which, two belonged to the conventional group, and one belonged to the DSG group. All three were excluded and replaced. Figure 3 shows the CONSORT flow diagram of patient selection and allocation.

### 3.2 Outcomes

**FIGURE 4:**
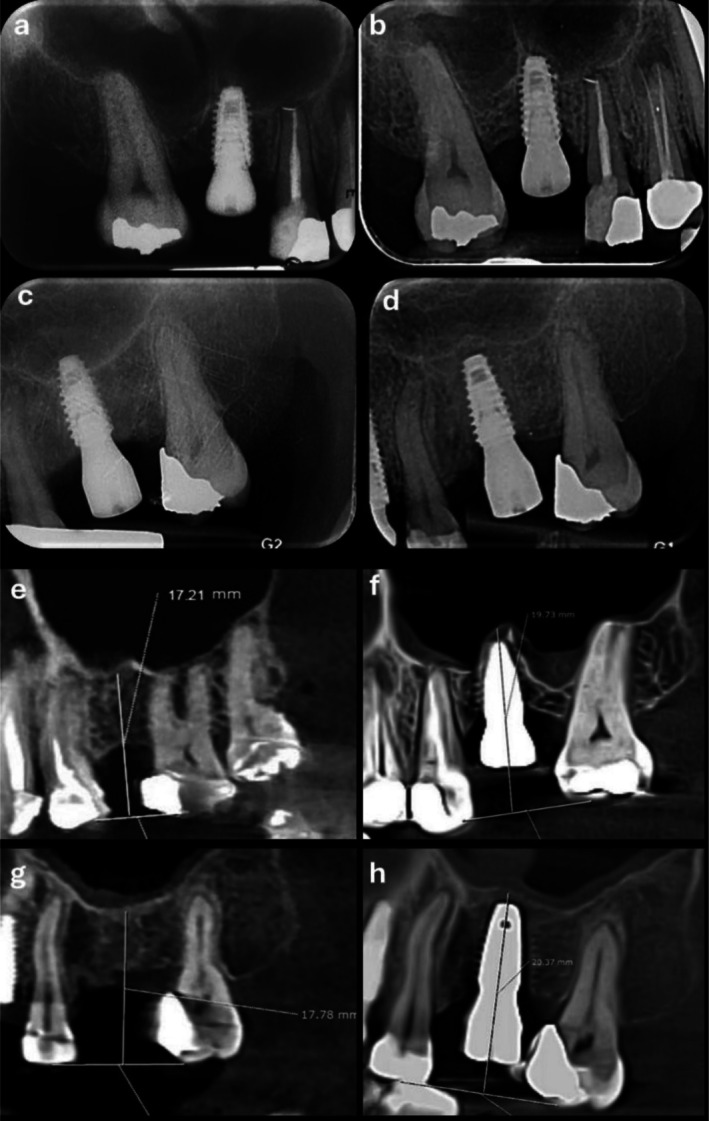
(a) Periapical radiograph in the conventional group (immediate after surgery), (b) periapical radiograph in the conventional group (6 months follow‐up), (c) periapical radiograph in the guided group (immediate after surgery), (d) periapical radiograph in the guided group (6 months follow‐up), (e) CBCT in the conventional group (before surgery), (f) CBCT in the conventional group (6 months follow‐up), (g) CBCT in the guided group (before surgery) and (h) CBCT in the guided group (6 months follow‐up).

**FIGURE 5:**
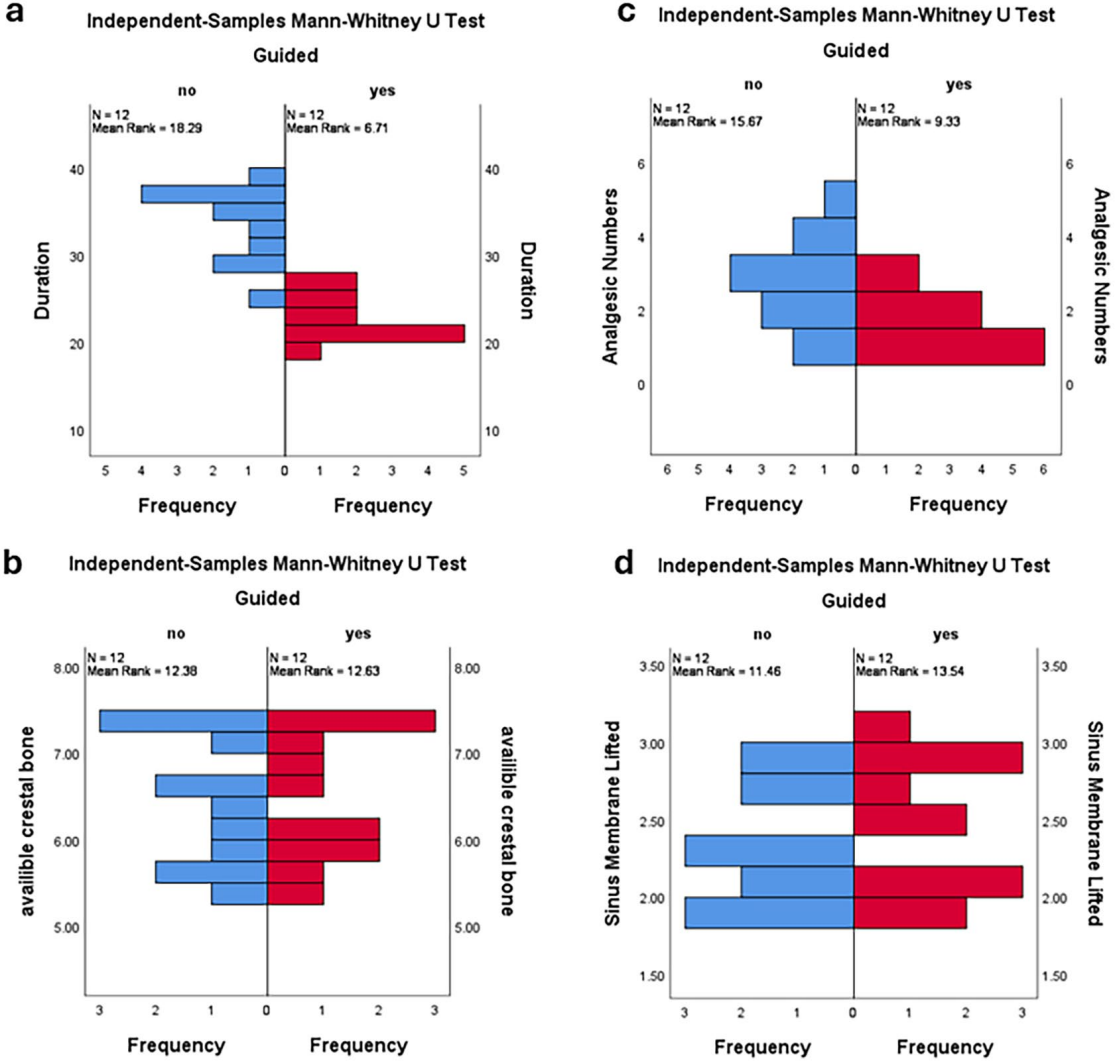
Graphs depicting (a) duration of the surgery, (b) available crestal bone, (c) number of analgesics taken and (d) lifted sinus membrane height.

**FIGURE 6:**
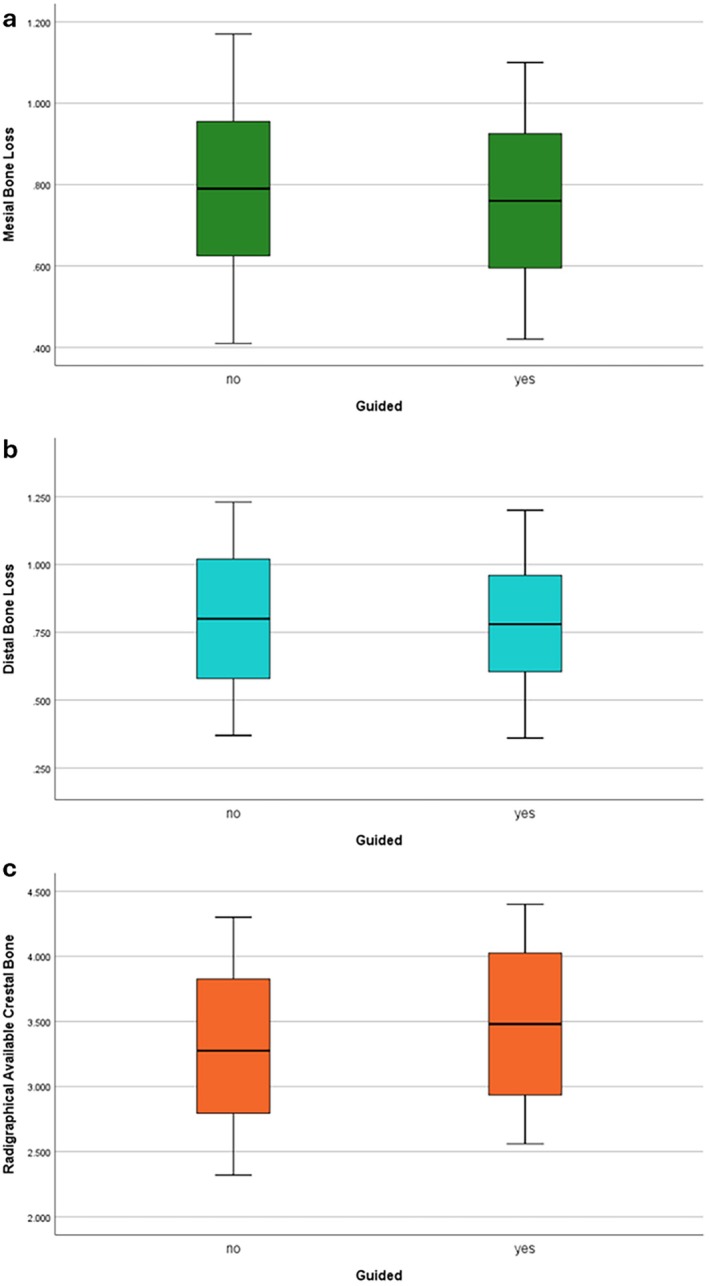
Boxplots indicate (a) mesial and (b) distal crestal bone loss and (c) available crestal bone amount.

Table 1 presents the study population and outcomes. Figure 4 depicts radiographical outcomes in two cases. The mean postoperative bone loss in the DSG group was 0.76 ± 0.21 mm in mesial and 0.78 ± 0.25 mm in distal aspects, while in the conventional group, these values were 0.79 ± 0.22 and 0.80 ± 0.27 mm, respectively. No significant differences were found between the groups for either the mesial (*p* = 0.744) or distal (*p* = 0.866) aspects. Initial mean bone height was 6.47 ± 0.74 mm in the DSG group and 6.45 ± 0.75 mm in the conventional group, with no significant difference (*p* = 0.932). The mean lift was 2.46 ± 0.41 in the DSG group and 2.35 ± 0.36 mm in the conventional group, also not significant (*p* = 0.47). The mean radiographic bone gain in the sinus was 3.48 ± 0.63 mm in the DSG group and 3.30 ± 0.65 mm in the conventional group, with no significant difference (*p* = 0.512). Figures 5 and 6 present bar charts and boxplots corresponding to the study outcomes. Further comparison between the DSG and conventional groups demonstrated a shorter operation time (22.33 ± 2.42 min vs. 33.17 ± 4.15 min, *p* < 0.001), fewer postoperative analgesics taken (1.67 ± 0.77 vs. 2.75 ± 1.21, *p* = 0.028), and smaller implant angulation deviation from the ideal position (2.97° ± 0.60° vs. 8.68° ± 1.38°; *p* < 0.001), all in favor of DSG. The mean mesiodistal deviation of the implant from the ideal position, however, was not significantly different between the two groups (0.54 ± 0.24 mm in the DSG group vs. 0.49 ± 0.29 mm in the conventional group, *p* = 0.652).

**TABLE 1:** Descriptive analysis of procedural variables.

Variable	Total (*N* = 24)		Guided (*N* = 12)		Conventional (*N* = 12)		*p*
	Mean	SD	Mean	SD	Mean	SD	
Duration	27.75	6.45	22.33	2.42	33.17	4.152	< 0.001
Analgesic number	2.21	1.14	1.67	0.77	2.75	1.215	0.028
Angulation	5.82	3.09	2.97	0.60	8.68	1.38	< 0.001
Mesiodistal distance	0.51	0.26	0.54	0.24	0.49	0.29	0.652
Available bone height	6.46	0.72	6.47	0.74	6.45	0.74	0.932
Sinus lift	2.40	0.38	2.46	0.41	2.35	0.36	0.47
Radiographic bone gain	3.39	0.63	3.48	0.63	3.30	0.65	0.512
Mesial bone loss	0.77	0.21	0.76	0.21	0.79	0.22	0.744
Distal bone loss	0.79	0.25	0.78	0.25	0.80	0.27	0.866

Three patients experienced sinus membrane perforation during surgery and were excluded from the outcome analysis as per protocol.

In the DSG group, 1 perforation was observed out of 13 cases (7.7%; 95% CI: 1.4%–33.3%), while in the crestal sinus lift group, 2 perforations occurred out of 14 cases (14.3%; 95% CI: 4.0%–39.9%). Fisher's exact test demonstrated no statistically significant difference in perforation rates between the two groups (odds ratio = 0.5; *p* = 1.0). These results suggest that, within the limitations of our sample size, the risk of perforation did not significantly differ between the DSG and crestal sinus lift techniques.

These cases have been added to the CONSORT flow diagram and are further discussed in Section 4.

A sensitivity analysis excluding the patient with two implants was conducted to evaluate the potential effect of clustering. The findings were consistent with the main analysis, with no changes in statistical significance across the outcomes (Table S2).

## 4 Discussion

In the present study, sinus membrane perforation occurred in 7.7% of cases in the DSG group and 14.3% in the conventional group; however, this difference was not statistically significant (*p* = 1.0). To the best of our knowledge, no previous study has compared the frequency of membrane perforation between these two specific techniques. Our results fall within the reported range for crestal sinus lift techniques in previous studies—from 2.2% to 21.4% in the systematic review by Romero‐Millán et al. (2012) and from 0% to 17% in the review by Molina et al. (2022). For the hydraulic SFE system, available data are limited, but previous clinical trials have reported perforation rates of 0% (Lopez et al. 2014) and 3.3% (Cho et al. 2017).

As observed in our results, the incidence of perforation was lower in the DSG group. We hypothesize that this may be due to the nature of the technique. Since the diameter of the drill should match the DSG sleeve's length, determination occurs more accurately, decreasing the risk of perforation. However, since the difference was not statistically significant, no definitive conclusion can be drawn from this observation.

The surgical time was significantly shorter in the DSG group than the conventional group in the present study, which was in agreement with the results of Vercruyssen et al. (2014), who used mucosa‐supported surgical guides. This finding is due to the elimination of flap elevation and its subsequent suturing in the DSG technique. Younes et al. (2019) found no significant difference in surgical time between the DSG and the conventional groups. This result was different from the present findings, which may be due to the fact that they defined the surgical time as the time spent for the fabrication of DSG plus the duration of surgery. The duration of surgery in the DSG group was significantly lower than that in the conventional group in their study.

Although the operative time was statistically shorter in the guided group, it is important to note that digital planning and guide fabrication require additional preoperative time and resources. Therefore, the overall treatment duration might not differ significantly. However, from the patient's perspective, only the surgical chair time is experienced, which was indeed shorter in the guided group. As such, while this reduction may have limited clinical impact for the clinician or technician, it could still be considered a clinically relevant advantage from the patient's point of view.

Assessment of postoperative pain according to the number of taken analgesics revealed a significantly lower level of pain in the DSG group. The same result was reported for the flapless surgical procedures in a systematic review by Romandini et al. (2023). The reason can be the shorter surgical time and edema, inflammation, and pain associated with flap elevation, and higher patient comfort in flapless surgery (Romandini et al. 2023). This difference is often magnified in extensive surgical procedures. Although the surgical site was limited in size in the present study, significantly higher postoperative pain in the conventional group can also be due to the use of an osteotome and mallet to break the sinus floor in this group, versus the hydraulic system used in the DSG group. The same was reported as the reason for significantly lower postoperative pain and discomfort in the DSG group in a study by Abo Elkasem et al. (2023). Although the number of painkillers taken was statistically lower in the guided group, the absolute difference between groups was relatively small. Therefore, the clinical relevance of this finding remains uncertain and should be interpreted with caution.

Crestal bone loss at the mesial and distal was not significantly different between the two groups of the present study, which was in agreement with the results of Pisoni et al. (2016). However, Lahoti et al. (2021) and Archana et al. (2023) reported significantly lower crestal bone loss in flapless surgery, compared with flap surgery (Magrin et al. 2020). The reason can be the preservation of the integrity of the periosteum, which is responsible for the provision of blood supply to the bone, in flapless surgery (Lahoti et al. 2021; Archana et al. 2023). In the current study, the extent of flap elevation was minimal since bone augmentation was not required. Also, the surgeon attempted to elevate the flap uniformly to minimize its traumatization, which resulted in faster healing. Moreover, all patients received one single implant, requiring flap retraction only for a short period of time; all these parameters minimized tissue traumatization and resulted in an insignificant difference in crestal bone loss between the two groups.

The difference between the two groups in the amount of newly formed bone (radiographical) in the sinus was not significant in the current study. In contrast, Abo Elkasem et al. (2023) reported significant superiority of the hydraulic technique to the osteotome technique in this regard; they used xenograft bone substitute following SFE in both groups. Hydraulic pressure can cause uniform separation of the Schneiderian membrane over a larger area due to liquid flow (Abo Elkasem et al. 2023), which provides a greater space for placement of bone substitutes compared with the osteotome technique and may result in greater new bone formation, as reported in their study. Since no bone substitute was used in the present study, the lack of a significant difference in new bone formation between the two groups may be justified.

In the present study, the deviation of mesiodistal implant angulation from the ideal position was smaller in the DSG group; these findings were in line with the split‐mouth randomized controlled trial by Magrin et al. (2020), where angular deviations were lower when digitally guided surgery was performed compared to conventional guided surgery. Angulation errors depend on several factors such as the experience and expertise of the surgeon, extent of the surgical site, and presence of adjacent teeth. DSG can be of great help, especially for novice surgeons. However, it should be noted that even the most accurate DSGs with the highest adaptation may have flaws, which explains the presence of some degree of deviation in the DSG group as well (Romandini et al. 2023).

Although the difference in mesiodistal angulation between the two groups was statistically significant, the mean deviation in both groups remained within clinically acceptable limits and did not compromise prosthetic planning or implant success. Therefore, the observed difference, while statistically relevant, may have limited clinical impact.

Deviation in mesiodistal distance from the ideal position was not significantly different between the two groups in the current study, which was in contrast to the results of previous investigations (Romandini et al. 2023; Magrin et al. 2020). The reason may be the limited size of edentulous area in the present study. Achieving ideal mesiodistal distance from adjacent teeth in limited edentulous areas is easier; while, larger edentulous areas were evaluated in their systematic review, which are more susceptible to errors, emphasizing on the benefits of DSGs for large edentulous areas. Similarly, a study by Chandran et al. (2023) comparing DSG versus free‐hand immediate implant placement reported that in the freehand group, crestal deviations of 1.13 ± 0.89 mm and 1.00 ± 0.76 mm were found in the mesiodistal and buccolingual directions, respectively, versus 0.34 ± 0.26 mm (*p* < 0.001) and 0.37 ± 0.24 mm (*p* = 0.03) in the static guided surgery group. When compared to our study, placement of immediate implant placement versus healed ridge may pose more technical challenges and therefore more prone to deviations, which may to some extent explain the difference in findings of two studies.

This study has several limitations. Postoperative pain was assessed based on the number of analgesics taken, which—although objective—may not fully capture patients' subjective experiences due to individual variability in pain perception and medication use. Additionally, no bone grafts were used during sinus floor elevation. While some studies suggest that graft omission may enhance implant survival in cases of minimal elevation, the lack of grafting could still affect the extent of new bone formation. Moreover, the reliability of the Valsalva maneuver for detecting membrane perforation in the flapless group may have been limited due to constrained visibility and potential bleeding at the surgical site. Another limitation of the study is the absence of statistical correction for multiple comparisons, which may increase the risk of type I error. Lastly, the relatively small sample size and short follow‐up period may limit the generalizability of the results. Future studies with larger cohorts, extended follow‐up durations, and direct comparisons between grafted and non‐grafted approaches are warranted, along with more robust statistical analysis.

## 5 Conclusion

Within the limitations of this study, the use of a digitally guided hydraulic crestal SFE technique may offer advantages over the conventional method, particularly in terms of postoperative discomfort, reduced surgical time, and improved implant angulation. However, the lack of statistically significant differences in some parameters and the relatively small sample size warrant cautious interpretation of the results. Further studies with larger samples and longer follow‐up are needed to validate these findings.

## Author Contributions

**Mahsa Enssi:** conceptualization, methodology, data curation, software, investigation, formal analysis, visualization, writing – original draft, writing – review and editing. **Mohammadreza Karimi:** conceptualization, methodology, investigation, writing – original draft, writing – review and editing, supervision. **Ardavan Etemadi:** conceptualization, methodology, investigation, writing – review and editing, writing – original draft, supervision. **Mahsa Khademi:** methodology, investigation, writing – review and editing. **Hamoun Sabri:** conceptualization, methodology, supervision, writing – review and editing.

## Conflicts of Interest

The authors declare no conflicts of interest.

## Supporting information


**Appendix S1:** clr70062‐sup‐0001‐AppendixS1.doc.

## Data Availability

The data that support the findings of this study are available from the corresponding author upon reasonable request.
